# The Effect of *Moringa oleifera* on Body Weight and Blood Pressure: A Systematic Review and Meta‐Analysis of Randomized Controlled Trials

**DOI:** 10.1002/fsn3.71899

**Published:** 2026-05-22

**Authors:** Mahnoush Mehrzad Samarin, Fatemeh Sheikhhossein, Amir Hossein Khalilkhaneh, Shadi Ghaemi, Amin Abbasi, Hadi Bazyar, Farhang Djafari, Zahra Dianati, Seyed Mojtaba Khodashenas, Gholamreza Askari, Mohammad Reza Amini

**Affiliations:** ^1^ Department of Physical Education and Sport Sciences, Science and Research Branch Islamic Azad University Tehran Iran; ^2^ Department of Clinical Nutrition, School of Nutritional Sciences and Dietetics Tehran University of Medical Sciences (TUMS) Tehran Iran; ^3^ Department of Community Nutrition, School of Nutritional Sciences and Dietetics Tehran University of Medical Sciences Tehran Iran; ^4^ Department of Food Science and Technology, Faculty of Nutrition and Food Sciences Tabriz University of Medical Sciences Tabriz Iran; ^5^ Department of Public Health Sirjan School of Medical Sciences Sirjan Iran; ^6^ Student Research Committee Sirjan School of Medical Sciences Sirjan Iran; ^7^ School of Health, Medical and Applied Sciences Central Queensland University Brisbane Australia; ^8^ Department of Clinical Nutrition & Dietetics, National Nutrition & Food Technology Research Institute Shahid Beheshti University of Medical Sciences Tehran Iran; ^9^ Student Research Committee, Department of Clinical Nutrition & Dietetics, National Nutrition & Food Technology Research Institute Shahid Beheshti University of Medical Sciences Tehran Iran; ^10^ Nutrition and Food Security Research Center Isfahan University of Medical Sciences Isfahan Iran

**Keywords:** body weight, hypertension, *Moringa oleifera*, obesity

## Abstract

*Moringa oleifera* is suggested as an antihypertensive and anti‐obesity agent, but there is insufficient evidence to establish a causal link between *Moringa oleifera* and reductions in blood pressure and body measurements in humans. This systematic review and meta‐analysis seek to examine the effects of *Moringa oleifera* on body weight and blood pressure. A search was conducted through Web of Science, Scopus, and PubMed, along with a manual review of bibliographies, for articles published up to July 2024. Out of 2295 articles, eight randomized controlled trials (RCTs) were included. The results indicated that *Moringa oleifera* consumption significantly reduced systolic blood pressure (SBP) by weighted mean differences (WMDs): −6.00 mmHg, *p* < 0.001 and diastolic blood pressure (DBP) by WMD: −7.32 mmHg, *p* < 0.001. However, no significant changes were observed in body mass index (BMI) (*p* = 0.435) and body weight (*p* = 0.938) compared to those who did not consume *Moringa oleifera* . Subgroup analyses revealed that SBP significantly decreased in adults consuming 5–10 g/day of *Moringa oleifera* , particularly in individuals with a BMI below 29.9 and those treated for more than 4 weeks. Furthermore, *Moringa oleifera* intake was linked to a significant reduction in DBP among participants taking < 10 g/day, those who were overweight, and individuals who supplemented for over 4 weeks. Our systematic review and meta‐analysis of RCT indicated that *Moringa oleifera* had a significant impact on blood pressure, but it did not affect body weight or BMI. Further research is necessary to confirm these findings.

## Graphical abstract

*Moringa oleifera* significantly reduced systolic and diastolic blood pressure but had no effect on body weight or BMI. Greater BP reductions were observed with 5–10 g/day intake in overweight individuals and interventions lasting more than 4 weeks.

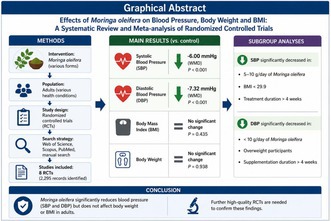

## 1 Introduction

Obesity and hypertension have emerged as major global health challenges over the past three decades (Chew et al. 2023; Zhang et al. 2024). According to the World Health Organization (WHO), in 2022, over 1 billion people—equivalent to one in eight individuals—were living with obesity (Ma et al. 2021). This alarming trend highlights the growing burden of metabolic disorders. The WHO also estimates that over 1.28 billion adults globally suffer from hypertension (Mills et al. 2020). Obesity and hypertension are influenced by multiple interconnected factors, including genetic predisposition (Kato 2012), dietary habits (Zhao et al. 2024), sedentary lifestyles (Park et al. 2020), and environmental influences (Lee et al. 2019). Diet is a key modifiable determinant of body weight and blood pressure (Lelong et al. 2019). Given the strong link between diet and cardiometabolic health, nutritional interventions remain a cornerstone in the management of obesity and hypertension (Gorostegi‐Anduaga et al. 2018). As part of these nutritional interventions, various natural products have gained attention as alternative or complementary therapies due to their antioxidant, anti‐inflammatory, and insulin‐sensitizing properties (Alves et al. 2019; Ammendola and Scotto d'Abusco 2022; Ghaffari and Roshanravan 2020).

Among these, *Moringa oleifera* Lam. is a fast‐growing perennial tree from the family Moringaceae that has been widely consumed as both food and traditional medicine across South Asia and Africa for centuries (Liu et al. 2022). It is a nutrient‐dense plant containing a diverse range of bioactive phytochemicals, including flavonoids, glucosinolates, carbamates, phenols, steroids, and carotenoids, which contribute to its antioxidant and anti‐inflammatory properties. Its phytochemical profile includes flavonoids such as quercetin and kaempferol derivatives (approximately 10–50 mg QE/g dry weight), along with phenolic acids like chlorogenic and caffeic acids, contributing to a total phenolic content of about 20–120 mg GAE/g dry weight. It also contains notable levels of glucosinolates, particularly glucomoringin (around 2–10 mg/g dry weight), as well as carotenoids including β‐carotene (roughly 15–30 mg/100 g dry weight) and phytosterols such as β‐sitosterol. In addition, bioactive peptides derived from its leaf proteins have recently attracted attention due to their potential biological activities. Together, these compounds are believed to play a key role in the plant's antioxidant and anti‐inflammatory effects (Liu et al. 2022). Ethnobotanically, *Moringa oleifera* has been traditionally used in the management of metabolic, cardiovascular, inflammatory, and infectious conditions (Liu et al. 2022). Research has shown that *Moringa oleifera* may have beneficial impacts on body weight regulation (Bais et al. 2014; Metwally et al. 2017; Redha et al. 2021) and blood pressure control (Okorie et al. 2019; Acuram and Chichioco Hernandez 2019; Aktar et al. 2019).

A limited number of meta‐analyses have been conducted to investigate this association. Kamrul Hasan et al. (2023) conducted a systematic review and meta‐analysis, finding that *Moringa oleifera* extract reduced blood pressure in type 2 diabetes and prediabetes patients, with no effect on body weight. However, their analysis included only two studies assessing blood pressure and body weight, limiting the reliability of the findings. Another study reviewed the antihypertensive properties of *Moringa oleifera* , attributing their effects to the inhibition of key enzymes in blood pressure regulation, including angiotensin‐converting enzyme, acetylcholinesterase, arginase, and phosphodiesterase 5 (Hassan et al. 2021). Results from another systematic review by Raja Kumar et al. supported the preventive effects of *Moringa oleifera* on obesity and hyperlipidemia (Raja Kumar et al. 2022). Findings from a systematic review examined the anti‐obesity effects of *Moringa oleifera* , highlighting bioactive compounds like quercetin, kaempferol, and isothiocyanates, which may inhibit adipogenesis through pathways such as AMPK, PI3K/AKT, and Wnt/β‐catenin (Andy Putra and Louisa 2023). Nevertheless, available reviews mainly rely on animal studies, limiting their clinical relevance. Moreover, no meta‐analysis has evaluated the effects of *Moringa oleifera* extract on blood pressure and body weight as primary outcomes in humans. Given these gaps, this study aims to conduct a systematic review and meta‐analysis of randomized controlled trials (RCTs) to explore the effects of *Moringa oleifera* on body weight and blood pressure.

## 2 Methods

### 2.1 Systematic Search and Study Selection

The study was conducted in accordance with the 2020 PRISMA guidelines (Page et al. 2021) (Table S1). A systematic literature search was conducted in Web of Science, Scopus, and the PubMed search engine up to July 27, 2024, with no restrictions on dates. The search was conducted by using medical subject headings (MeSH) and non‐MeSH keywords as follows: (*Moringa*[tiab] OR “ *Moringa oleifera* ”[tiab] OR “Drumstick tree”[tiab] OR “ *Moringa oleifera* ”[Mesh]) AND (Intervention[tiab] OR RCT[tiab] OR Randomized[tiab] OR Placebo[tiab] OR Assignment[tiab] OR Trial[tiab] OR Trials[tiab] OR Randomised[tiab] OR “Methods”[Mesh] OR “Double‐Blind”[tiab] OR “Controlled Clinical Trial”[Publication Type] OR “Randomized Controlled Trial”[Publication Type] OR “Placebos”[Mesh] OR “Clinical Trial”[Publication Type] OR “Placebo Effect”[Mesh] OR “Clinical Trials as Topic”[Mesh] OR “Double‐Blind Method”[Mesh] OR “Cross‐Over Studies”[Mesh]). A further literature search was conducted using Google Scholar, which screened *Moringa oleifera* related terms up to July 27, 2024. The first 10 pages of all search records were scanned. Only English‐language studies were included in the search strategy. Two authors, F.S. and A.H.K. (independently), conducted systematic screening. Any disagreements were resolved through discussions with another researcher (M.R.A.).

### 2.2 Experimental Methods

Two authors assessed the titles, abstracts, and full texts of qualifying studies independently (F.S. and A.H.K.) based on the PICOS (population/intervention/comparison/outcome/study design) framework: P (healthy adults and subjects with prediabetes, diabetes, HIV, and hyperlipidemia), I ( *Moringa oleifera* ), C (control group received placebo includes capsule and the already prepared diet), O (body weight, body mass index [BMI], and blood pressure), and S (parallel clinical trial). Original research was chosen for inclusion if it (1) had a parallel RCT design, (2) conducted on adult population (age > 18 years), (3) reported the effects of dietary *Moringa oleifera* supplementation on body weight, BMI, and blood pressure with a control group; (4) and administered *Moringa oleifera* for at least 2 weeks. Studies were excluded if *Moringa oleifera* was in combination with other supplements, they were uncontrolled studies, animal and in vitro studies, reviews, editorials, and studies without sufficient data on related consequences.

### 2.3 Data Extraction

Data extraction and study selection were carried out separately by two investigators (F.S. and A.H.K.). Any disputes between reviewers were resolved through discussion with the third author. The following attributes were recorded for every study that qualified: first author, publication year, country, study design, gender, target population, sample size in intervention and control groups, mean age and BMI of participants in intervention and control groups, dose and duration of intervention, type of *Moringa oleifera* , and outcomes. If studies reported any other effect sizes or various units for body weight, BMI, and blood pressure, we converted to the same unit and effect size (means ± standard deviation [SDs]).

### 2.4 Quality Assessment

Two different writers (F.S. and A.H.K.) used a Cochrane Collaboration method to evaluate the quality of the included papers (Higgins et al. 2011). Based on this Handbook, each item has a low risk of bias, a high risk of bias, or an unclear risk of bias. The overall risk of bias for a study was classified as good, fair, or weak (Cumpston et al. 2019).

### 2.5 Statistical Analyses

We calculated weighted mean differences (WMDs) and 95% confidence intervals (CIs) to estimate *Moringa oleifera* effects on body weight, BMI, systolic blood pressure (SBP), and diastolic blood pressure (DBP) with use of random effects analysis (DerSimonian and Laird 1986). To calculate the pooled effect sizes, we used the mean change and SD of each outcome in the *Moringa oleifera* and control groups with the use of the approach described by Higgins et al. (2011). When studies reported standard errors (SEs), interquartile ranges (IQRs), or 95% CIs rather than SDs, related statistical conversions were performed to calculate SD values (Hozo et al. 2005). When intervention arms shared a common control group, the sample size of the control group was evenly divided. We conducted subgroup analyses based on study duration (> 4 or < 4 weeks), dose range (≤ 5, 5–10, and ≥ 10 g/day), and BMI range (< 25, 25–29.9, and ≥ 30 kg/m^2^). We utilized sensitivity analysis to assess the impact of each RCT on the final results (Chandler et al. 2019). Begg's (Begg and Mazumdar 1994) and Egger's (Egger et al. 1997) tests were used to examine publication bias. Moreover, we applied Cochran's *Q* test and *I*‐squared (*I* ^2^) statistic to find out heterogeneity between the studies (Chandler et al. 2019). We considered significant heterogeneity between study if *p* values < 0.05 and *I* ^2^ > 50%. Also, *p* value < 0.05 was considered statistically significant. We applied STATA software, version 14.0 (StataCorp, College Station, TX, USA) for conducting all tests.

## 3 Results

### 3.1 Literature Search

**FIGURE 1:**
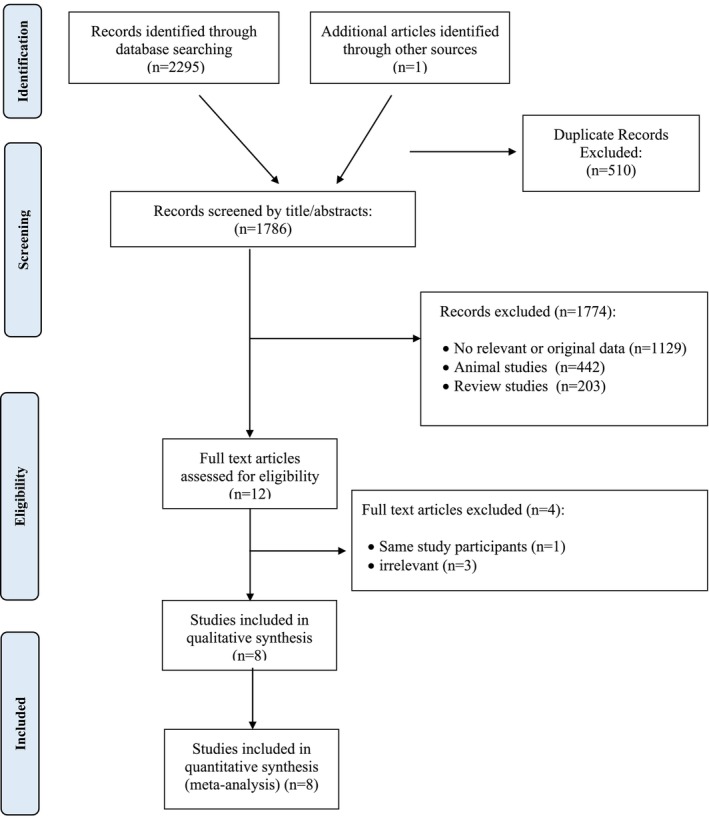
Flowchart of the number of studies identified and selected into the meta‐analysis.

The study's identification procedure flow diagram is shown in Figure 1. 2295 records were initially discovered using manual search and all electronic databases. Also, one article was identified through other sources. Then, screening based on title or abstract helped to remove 1774 irrelevant studies and 510 duplication records. Among the 12 remaining publications, four articles were excluded after reading the full text, since they did not have a relevant outcome (*n* = 3), and in one study (Gambo et al. 2021) there were participants in common with Gambo and Gqaleni (2022) study. Overall, eight studies (Sarfraz et al. 2023; Afiaenyi et al. 2025; Hameed et al. 2023; Gambo and Gqaleni 2022; Díaz‐Prieto et al. 2022; Tshingani et al. 2017; Taweerutchana et al. 2017; Sandoval and Jimeno 2013) had the eligibility criteria for the final analysis. The study of Afiaenyi et al. (2025) had three effect sizes (a, b, and c) because the intervention groups were divided into three supplementation groups. Also, the study of Hameed et al. (2023) had two effect sizes (a and b).

### 3.2 Study Characteristics

Table 1 provides an overview of the general features of these RCTs. Included trials were published between 2013 and 2023. A total of 588 individuals with a mean age of 26–60 years are included in the present Systematic Review and Meta‐analysis. All studies applied parallel design and both genders enrolled in them. The Control group was fed diets without *Moringa oleifera* (Afiaenyi et al. 2025; Tshingani et al. 2017), placebo supplement (Hameed et al. 2023; Gambo and Gqaleni 2022; Díaz‐Prieto et al. 2022; Taweerutchana et al. 2017; Sandoval and Jimeno 2013), and placebo supplement + 10 mg atorvastatin (Sarfraz et al. 2023). The dosage of *Moringa oleifera* ranged from 2 to 60 g/day, and the intervention duration varied between 2 and 24 weeks. These trials were conducted in Nigeria (Afiaenyi et al. 2025), Pakistan (Sarfraz et al. 2023; Hameed et al. 2023), Spain (Díaz‐Prieto et al. 2022), South Africa (Gambo and Gqaleni 2022), Philippines (Sandoval and Jimeno 2013), Thailand (Taweerutchana et al. 2017), and Congo (Tshingani et al. 2017). Also, studies are carried out in healthy adults (Sandoval and Jimeno 2013) and subjects with prediabetes (Díaz‐Prieto et al. 2022), diabetes (Afiaenyi et al. 2025; Hameed et al. 2023; Taweerutchana et al. 2017), HIV (Gambo and Gqaleni 2022; Tshingani et al. 2017), and hyperlipidemia (Sarfraz et al. 2023).

**TABLE 1:** Demographic characteristics of the included studies.

First author (year)	Location	Study design	Health status	Sex	Sample size	Duration (week)	Mean age (year)	Baseline BMI (kg/m^2^)	Intervention group	Comparator group	Outcome
1. Afiaenyi (a) (2025)	Nigeria	RCT	T2D	Both	20	2	59.55	23.98	20 g *Moringa* leaves	Control group was fed diets without *Moringa oleifera* leaves	SBP/DBP
2. Afiaenyi (b) (2025)	Nigeria	RCT	T2D	Both	20	2	60.45	24.22	40 g *Moringa* leaves	Control group was fed diets without *Moringa oleifera* leaves	SBP/DBP
3. Afiaenyi (c) (2025)	Nigeria	RCT	T2D	Both	20	2	59.4	24.3	60 g *Moringa* leaves	Control group was fed diets without *Moringa oleifera* leaves	SBP/DBP
4. Díaz‐Prieto (2022)	Spain	RCT	Prediabetes	Both	65	12	55.1	27.25	2.4 g *Moringa oleifera* Lam. leaves	Placebo	SBP/DBP
5. Gambo (2022)	South Africa	RCT	People living with HIV that are on antiretroviral therapy	Both	177	24	41.57	24.29	15 g *Moringa oleifera* Lam. leaf	Placebo	Weight/BMI
6. Hameed (a) (2023)	Pakistan	RCT	T2D	Both	48	12	55.5	27.5	3 g *Moringa oleifera* Lam. leaf	Placebo	Weight/SBP/DBP
7. Hameed (b) (2023)	Pakistan	RCT	T2D	Both	48	12	54	28.5	6 g *Moringa oleifera* Lam. leaf	Placebo	Weight/SBP/DBP
8. Sandoval (2013)	Philippines	RCT	Healthy	Both	68	30 day	37.91	24.28	2.1 g Malunggay ( *Moringa oleifera* )	Placebo	Weight/BMI
9. Sarfraz (2023)	Pakistan	RCT	Hyperlipidemic patients	Both	30	45 day	25–55	33.4	2 g *Moringa oleifera* + 40 mg atorvastatin	Placebo + 10 mg atorvastatin	BMI
10. Taweerutchana (2017)	Thailand	RCT	T2D	Both	32	4	55	27.6	8 g *Moringa oleifera* leaf	Placebo	Weight/SBP/DBP
11. Tshingani (2017)	Democratic Republic of the Congo	RCT	HIV‐infected patients	Both	60	24	48.25	21.6	30 g *Moringa oleifera* Lam. leaf	Received nutritional counseling	BMI

Abbreviations: BMI, body mass index; DBP, diastolic blood pressure; RCT, randomized controlled trial; SBP, systolic blood pressure; T2D, type 2 diabetes.

### 3.3 Risk of Bias Assessment

The included studies' methodological quality differed by domain, according to the Risk of Bias assessment developed by the Cochrane Collaboration. Random sequence creation, allocation concealment, selective reporting, and other types of bias were found to have a low risk of bias in the majority of research. However, missing outcome data in several trials and participant and staff blinding were found to have a high risk of bias. Table 2 summarizes the details of the risk of bias of each study.

**TABLE 2:** Risk of bias for randomized controlled trials, assessed according to the Revised Cochrane risk‐of‐bias tool for randomized trials (RoB 1).

Publications	Random sequence generation	Allocation concealment	Selective reporting	Blinding (participants and personnel)	Blinding (outcome assessment)	Incomplete outcome data	Other source of bias
1. Afiaenyi (2025)	L	L	L	L	H	L	L
2. Díaz‐Prieto (2022)	L	L	L	L	H	H	L
3. Gambo (2022)	L	L	L	L	L	H	L
4. Hameed (2023)	L	U	L	H	H	L	L
5. Sandoval (2013)	L	L	L	L	H	H	L
6. Sarfraz (2023)	L	L	L	H	H	L	L
7. Taweerutchana (2017)	L	L	L	H	H	L	L
8. Tshingani (2017)	L	L	L	H	H	L	L

Abbreviations: H, high risk of bias; L, low risk of bias; U, unknown.

### 3.4 Effect of *Moringa oleifera* Leaf on Body Weight

**FIGURE 2:**
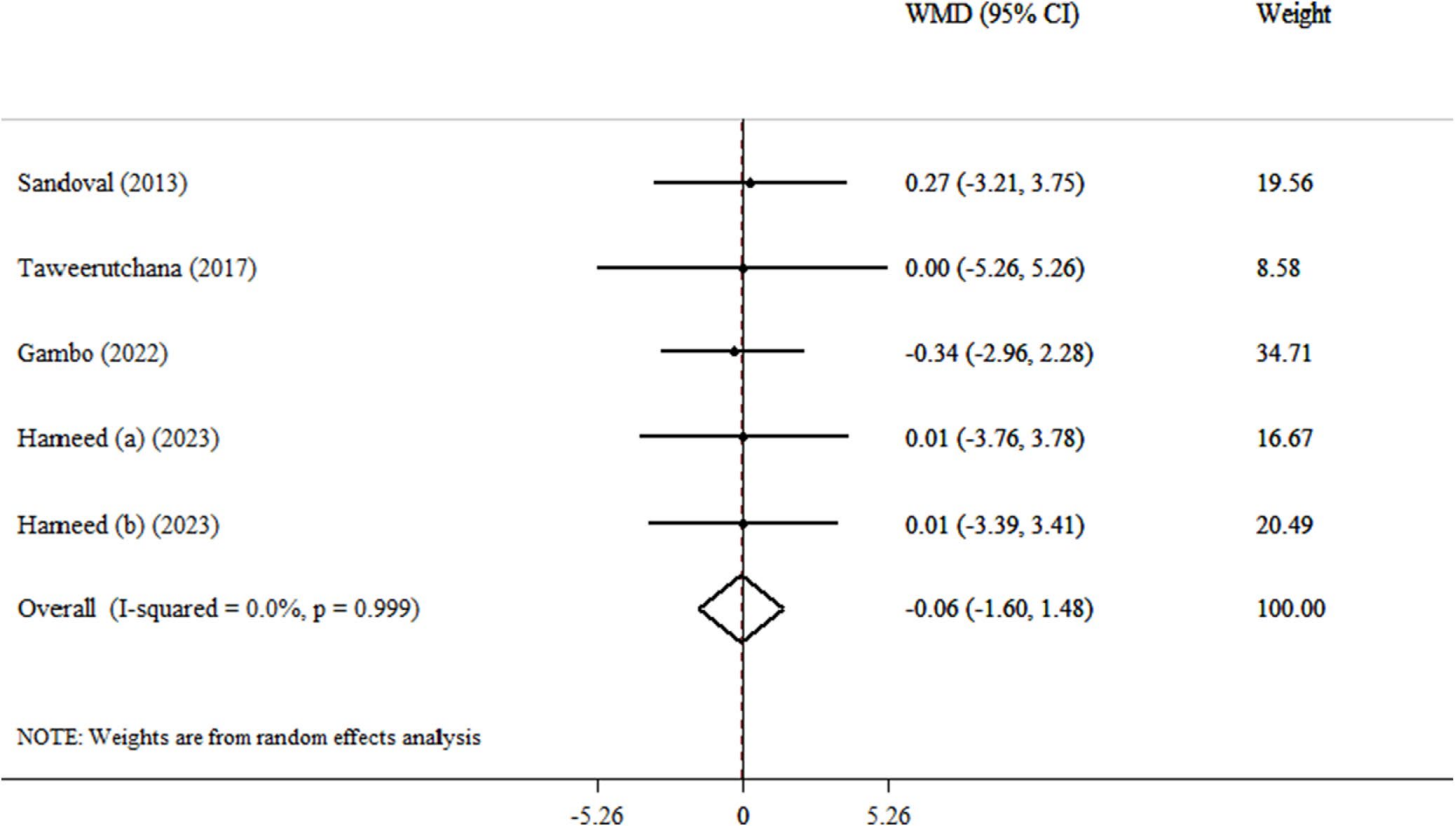
Forest plot detailing weighted mean difference and 95% confidence intervals (CIs) for the effect of *Moringa oleifera* on weight.

Four clinical trials and five effect size pooled findings demonstrated that *Moringa oleifera* did not have a discernible impact on body weight (WMDs: −0.06 kg; 95% CI: −1.60, 1.48; *p* = 0.938) (Hameed et al. 2023; Gambo and Gqaleni 2022; Taweerutchana et al. 2017; Sandoval and Jimeno 2013) (Figure 2). Also, heterogeneity was not observed between the included trials (*I* ^2^ = 0.0%, *p* = 0.999). Furthermore, the pooled results were unaffected by the subgroup analysis (Table 3). In the sensitivity analysis, each trial was gradually eliminated from the pooled analysis to evaluate the effect of each study on the pooled effect size. The results showed that eliminating each trial did not significantly change the WMD. This indicated that the meta‐analysis results remained constant and were not affected by any of the five effect sizes. Furthermore, we examined publication bias by using Begg's (*p* = 1.000) and Egger's test (*p* = 0.304).

**TABLE 3:** Subgroup analysis of included randomized controlled trials in meta‐analysis of the effect of *Moringa oleifera* leaf extract on body weight and blood pressure.

Group	No. of effect size	WMD (95% CI)	*p*	*I* ^2^ (%)	*p* heterogeneity	*p* for between subgroup heterogeneity
Weight						
Pooled effect size	5	−0.06 (−1.60, 1.48)	0.94	0.0	0.999	—
Duration (week)						
≤ 4	2	0.19 (−2.72, 3.75)	0.90	0.0	0.93	0.843
> 4	3	−0.16 (−1.98, 1.66)	0.86	0.0	0.98	
Dose (g)						
≤ 5	2	0.15 (−2.41, 2.71)	0.90	0.0	0.92	0.965
5–10	2	0.01 (−2.85, 2.86)	0.99	0.0	0.99	
≥ 10	1	−0.34 (−2.96, 2.28)	0.80	—	—	
Mean BMI						
< 25	2	−0.12 (−2.21, 1.97)	0.91	0.0	0.78	0.935
25–29.9	3	0.01 (−2.27, 2.29)	0.99	0.0	1.00	
BMI						
Pooled effect size	4	0.36 (−0.55, 1.28)	0.43	47.0	0.13	—
Duration (week)						
≤ 4	1	0.12 (−1.11, 1.35)	0.85	—	—	0.753
> 4	3	0.45 (−0.97, 1.86)	0.53	64.0	0.06	
Dose (g)						
≤ 5	2	0.00 (−1.16, 1.16)	1.00	0.0	0.57	0.564
≥ 10	2	0.67 (−1.01, 2.35)	0.43	80.0	0.02	
Mean BMI						
< 25	3	0.46 (−0.55, 1.48)	0.37	61.2	0.08	0.478
≥ 30	1	−0.93 (−4.36, 2.50)	0.60	—	—	
SBP						
Pooled effect size	7	−6.00 (−7.91, −4.09)	< 0.001	0.0	0.48	—
Duration (week)						
≤ 4	4	−4.68 (−10.97, 1.62)	0.14	0.0	0.40	0.665
> 4	3	−6.04 (−8.29, −3.8)	< 0.001	15.5	0.31	
Dose (g)						
≤ 5	2	−4.95 (−9.54, −0.36)	0.03	51.0	0.15	0.196
5–10	2	−6.73 (−9.29, −4.17)	< 0.001	0.0	0.93	
≥ 10	3	7.53 (−8.21, 23.27)	0.35	0.0	0.90	
Mean BMI						
< 25	2	−5.52 (−8.44, −2.59)	< 0.001	51.0	0.15	0.090
25–29.9	2	−6.73 (−9.29, −4.17)	< 0.001	0.0	0.93	
≥ 30	3	7.53 (−8.21, 23.27)	0.35	0.0	0.90	
DBP						
Pooled effect size	7	−7.32 (−9.23, −5.41)	< 0.001	2.0	0.41	—
Duration (week)						
≤ 4	4	−5.83 (−11.41, −0.25)	0.04	12.4	0.33	0.852
> 4	3	−7.42 (−9.83, −5.02)	< 0.001	24.8	0.26	
Dose (g)						
≤ 5	2	−6.80 (−10.49, −3.12)	< 0.001	48.8	0.16	0.128
5–10	2	−8.72 (−11.51, −5.93)	< 0.001	0.0	0.89	
≥ 10	3	1.04 (−8.50, 10.58)	0.83	0.0	0.98	
Mean BMI						
< 25	3	1.04 (−8.50, 10.58)	0.83	0.0	0.98	0.079
25–29.9	4	−7.67 (−9.58, −5.75)	< 0.001	0.3	0.39	

Abbreviations: LDL, low‐density lipoprotein; TC, total cholesterol; TG, triglycerides; WMD, weight mean difference.

### 3.5 Effect of *Moringa oleifera* Leaf on BMI

**FIGURE 3:**
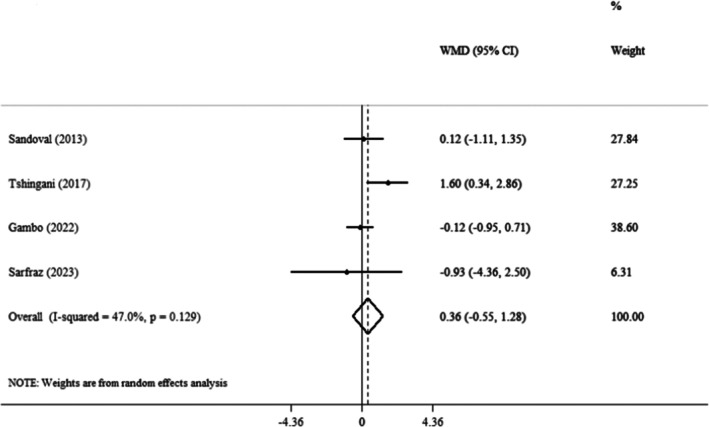
Forest plot detailing weighted mean difference and 95% confidence intervals (CIs) for the effect of *Moringa oleifera* on BMI.

Four clinical trials' pooled findings demonstrated that *Moringa oleifera* did not have a discernible impact on BMI (WMDs: 0.36 kg/m^2^; 95% CI: −0.55, 1.28; *p* = 0.435) (Sarfraz et al. 2023; Gambo and Gqaleni 2022; Tshingani et al. 2017; Sandoval and Jimeno 2013) (Figure 3). Also, insignificant heterogeneity was observed between the included trials (*I* ^2^ = 47%, *p* = 0.129). Furthermore, the pooled results were unaffected by the subgroup analysis (Table 3). In the sensitivity analysis, each trial was gradually eliminated from the pooled analysis to evaluate the effect of each study on the pooled effect size. The results showed that eliminating each trial did not significantly change the WMD. This indicated that the meta‐analysis results remained constant and were not affected by any of the four trials. Furthermore, we examined publication bias by using Begg's (*p* = 0.734) and Egger's test (*p* = 0.957).

### 3.6 Effect of *Moringa oleifera* Leaf on SBP

**FIGURE 4:**
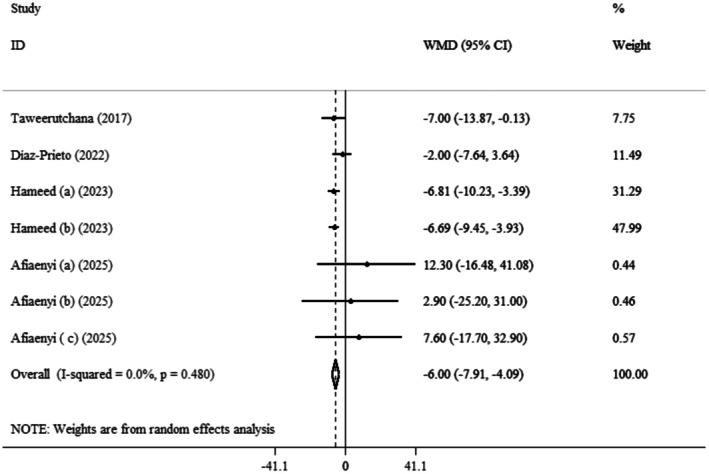
Forest plot detailing weighted mean difference and 95% confidence intervals (CIs) for the effect of *Moringa oleifera* on SBP.

Pooled data from four clinical trials with seven effect sizes demonstrated that *Moringa oleifera* significantly decreased SBP (WMDs: −6.00 mmHg; 95% CI: −7.91, −4.09; *p* < 0.001) (Afiaenyi et al. 2025; Hameed et al. 2023; Díaz‐Prieto et al. 2022; Taweerutchana et al. 2017) (Figure 4). Also, heterogeneity was not observed between the included trials (*I* ^2^ = 0.0%, *p* = 0.480). According to subgroup analysis, *Moringa oleifera* use can more effectively lower SBP in the subset of duration > 4 weeks (WMDs: −6.04 mmHg; 95% CI: −8.29, −3.80; *p* < 0.001), dose range between 5 and 10 g/day (WMDs: −6.73 mmHg; 95% CI: −9.29, −4.17; *p* < 0.001), and BMI range between 25 and 29.9 (WMDs: −6.73 mmHg; 95% CI: −9.29, −4.17; *p* < 0.001) (Table 3).

In the sensitivity analysis, each trial was gradually eliminated from the pooled analysis to evaluate the effects of each study on the pooled effect size. The results showed that eliminating each trial did not significantly change the WMD. This indicated that the meta‐analysis results remained constant and were not affected by any of the seven effect sizes. No publication bias was identified by using Begg's test (*p* = 0.133), but the result of Egger's test was contrary to Begg's test (*p* = 0.03).

### 3.7 Effect of *Moringa oleifera* Leaf on DBP

**FIGURE 5:**
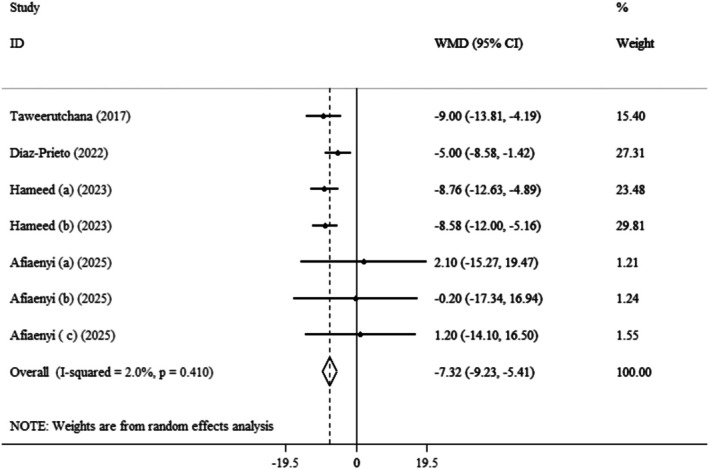
Forest plot detailing weighted mean difference and 95% confidence intervals (CIs) for the effect of *Moringa oleifera* on DBP.

Pooled data from four clinical trials with seven effect sizes demonstrated that the *Moringa oleifera* significantly decreased DBP (WMDs: −7.32 mmHg; 95% CI: −9.23, −5.41; *p* < 0.001) (Afiaenyi et al. 2025; Hameed et al. 2023; Díaz‐Prieto et al. 2022; Taweerutchana et al. 2017) (Figure 5). Also, insignificant heterogeneity was observed between the included trials (*I* ^2^ = 2.0%, *p* = 0.410). According to subgroup analysis, *Moringa oleifera* use can more effectively lower DBP in the subset of duration > 4 weeks (WMDs: −7.42 mmHg; 95% CI: −9.83, −5.02; *p* < 0.001), dose range between 5 and 10 g/day (WMDs: −8.72 mmHg; 95% CI: −11.51, −5.93; *p* < 0.001), and BMI range between 25 and 29.9 (WMDs: −7.67 mmHg; 95% CI: −9.58, −5.75; *p* < 0.001) (Table 3).

In the sensitivity analysis, each trial was gradually eliminated from the pooled analysis to evaluate the effects of each study on the pooled effect size. The results showed that eliminating each trial did not significantly change the WMD. This indicated that the meta‐analysis results remained constant and were not affected by any of the seven effect sizes. Furthermore, we examined publication bias using Begg's (*p* = 0.368) and Egger's test (*p* = 0.116).

## 4 Discussion

### 4.1 Main Findings

Interest in *Moringa oleifera* for metabolic health is increasing, but its effects on body measurements and blood pressure have not been extensively studied. This systematic review and meta‐analysis aimed to examine how *Moringa* influences body weight and blood pressure in adults. The findings showed that *Moringa* consumption led to significant reductions in SBP and DBP, while no significant changes were observed in body weight and BMI overall. Subgroup analyses indicated that SBP significantly decreased among adults taking 5–10 g/day of *Moringa*, in individuals with a BMI of < 29.9, and participants treated for more than 4 weeks. Additionally, *Moringa* intake was associated with a notable reduction in DBP in participants consuming < 10 g/day, those who were overweight, and individuals who took supplements for longer than 4 weeks.

Consistent with our findings, Afiaenyi et al. observed that a daily intake of 40 g of *Moringa* leaves influenced either systolic or diastolic blood pressure. In contrast, dosages of 20 and 60 g/day did not affect blood pressure levels (Afiaenyi et al. 2025). Another research study found that administering 6 mg of *Moringa* daily for 3 months resulted in lower diastolic and systolic blood pressure (Hameed et al. 2023). On the other hand, a recent article showed that a daily dose of 2.4 g of *Moringa* for 8 weeks did not lead to any changes in blood pressure (Díaz‐Prieto et al. 2022). Furthermore, research by Taweerutchana et al. (2017) indicated that taking 8 g/day of Moringa leaf capsules (compared to a placebo) over 4 weeks had no significant effect on either blood pressure measurement.

### 4.2 Bioactive Component

The food environment presents another promising opportunity for preventing and managing hypertension. Medicinal plants, which have been utilized in traditional medicine for centuries, contain bioactive compounds that may assist in regulating blood pressure. These plants are cost‐effective, readily available, and generally have few side effects, making them an appealing choice, particularly in developing countries. Among these plants, *Moringa oleifera* , commonly grown in tropical and subtropical regions, has attracted attention for its nutritional and medicinal benefits (Leone et al. 2016, 2015). Its leaves are particularly rich in proteins, minerals such as potassium, calcium, magnesium, and iron, as well as vitamins like β‐carotene and α‐tocopherol, dietary fiber, and bioactive compounds (Nova et al. 2020; Brilhante et al. 2017; Barichella et al. 2019). Because of this diverse nutrient profile, *Moringa* leaves are frequently used in traditional medicine in developing countries to address a range of health issues, including diabetes, cardiovascular diseases, and hypertension (Abdull Razis et al. 2014; Fahey 2005; Matic et al. 2018; Anwar et al. 2007; Abd El Latif et al. 2014). Acuram and Chichioco Hernandez (2019) investigated the antihypertensive effects of *Moringa oleifera* leaves in female mice. By the end of the treatment, the extracts significantly lowered blood pressure, aligning it with that of the control group. No significant differences were found between the high and low doses of the extracts (Acuram and Chichioco Hernandez 2019). In a separate study, Chen et al. explored the short‐term and long‐term effects of *Moringa oleifera* in rats with pulmonary hypertension induced by monocrotaline injection (60 mg/kg). The acute administration of 4.5 mg/kg of the extract reduced pulmonary arterial pressure to about 80% of the level observed in the control group (Chen et al. 2012).

### 4.3 Antihypertensive Mechanism

Multiple studies have confirmed the antihypertensive effects of *Moringa oleifera* , particularly its leaves. However, there is limited evidence regarding the effects of other parts, such as the seeds and pods. Additionally, the antihypertensive effects seem to be more pronounced in hypertensive animal models, while results in healthy animals vary. Animal model studies have shown that *Moringa oleifera* influences blood pressure through multiple mechanisms. The leaf extract promotes vasodilation by activating endothelial nitric oxide synthase (eNOS), thereby increasing nitric oxide (NO) availability. This eNOS activation is associated with the upregulation of protein kinase B (Akt) and a reduction in arginase‐1 expression (Aekthammarat, Tangsucharit, et al. 2020; Randriamboavonjy et al. 2019). Additionally, preclinical evidence showed that *Moringa oleifera* may reduce oxidative stress, further enhancing NO availability, which leads to the relaxation of vascular smooth muscle cells (VSMCs) via the activation of soluble guanylate cyclase (sGC) and the subsequent production of cyclic guanosine monophosphate (cGMP), a key mediator of vasodilation (Randriamboavonjy et al. 2019, 2017).

Moreover, *Moringa oleifera* induces vasodilation not only through NO but also via endothelium‐derived hyperpolarizing factors (EDHFs). These factors open potassium (K^+^) channels in VSMCs, resulting in membrane hyperpolarization and reduced calcium entry, ultimately decreasing VSMC contraction. The extract also exerts direct effects on VSMCs by inhibiting calcium (Ca^2+^) influx and mobilization, which contributes to a reduction in vascular tone and contractility by blocking voltage‐operated calcium channels (VOCCs) and receptor‐operated calcium channels (ROCCs) (Aekthammarat, Patchareewan, and Tangsucharit 2020).

Another proposed mechanism resulting from preclinical studies for the antihypertensive effects of *Moringa oleifera* involves the inhibition of the renin–angiotensin–aldosterone system (RAAS). Several studies have examined the angiotensin‐converting enzyme (ACE) inhibitory effects of various protein fractions and phenolic compounds present in the plant. One study found that protein hydrolysates from *Moringa oleifera* leaves significantly inhibited angiotensin‐converting enzyme (ACE) and renin, with maximum inhibition rates of 84.71% and 43.72%, respectively, for peptides under 1 kDa. Two bioactive peptides, Leu‐Gly‐Phe‐Phe (LGF) and Gly‐Leu‐Phe‐Phe (GLFF), reduced blood pressure in spontaneously hypertensive rats when administered orally, with the strongest effect seen 6 h post‐administration (Acuram and Chichioco Hernandez 2019).

Another study assessed the ACE inhibitory activity of methanolic and ethyl acetate extracts of *Moringa oleifera* (Ma et al. 2021). The ethyl acetate extract showed the highest activity and was biofractionated, leading to the isolation of quercetin‐3‐O‐glucoside and kaempferol‐3‐O‐glucoside. Quercetin‐3‐O‐glucoside exhibited significant ACE inhibition, reaching 75.74% at 28 μg/mL, while the activity of kaempferol‐3‐O‐glucoside could not be evaluated due to low yield (Ma et al. 2021). *Moringa oleifera* is recognized for its abundant bioactive compounds, such as β‐carotene, phenolic acids, flavonoids, and isothiocyanates, which provide significant antioxidant benefits (Tao et al. 2022). Research shows that the leaf extract reduces oxidative stress by neutralizing excessive reactive oxygen species (ROS), which contribute to cellular damage and chronic inflammation. This antioxidant effect is crucial for cardiovascular health since oxidative stress is a major factor in hypertension and cardiovascular diseases. Additionally, *Moringa oleifera* contains several hypotensive compounds, primarily thiocarbamates and isothiocyanates, which significantly contribute to lowering blood pressure (Chiș et al. 2024). Certain phenolic compounds and sterols also play a role in this hypotensive activity, though their effectiveness varies based on the specific part of the plant used and the type of extract employed.

### 4.4 Weight Management

In terms of weight management, Sandoval and Jimeno (2013) showed that 30 days supplementation with *Moringa* did not alter the body weight and BMI. Another study indicated that an intervention of 45 days with 2 g/day *Moringa* has no effect on BMI (Sarfraz et al. 2023). *Moringa oleifera* may support weight management through its nutrient density (Fahey 2005), metabolism‐boosting effects (Gopalakrishnan et al. 2016), appetite regulation, fat reduction (Sreelatha and Padma 2009), and antioxidant and anti‐inflammatory properties (Saini et al. 2016; Upadhyay et al. 2015). However, further clinical trials are needed to fully understand *Moringa oleifera* 's efficacy and mechanisms in weight management in humans (Redha et al. 2021). Conflicting results regarding its effects on weight and blood pressure management may arise from several factors, including variations in dosage, participant characteristics (such as age and health status), and differences in study design. The form of *Moringa* used (leaf powder, extract, or capsule) may also affect its effectiveness, as can participants' dietary habits and lifestyle factors (Stohs and Hartman 2015). These complexities highlight the need for more standardized studies to clarify *Moringa*'s role in weight management.

### 4.5 Strengths and Limitations

This systematic review and meta‐analysis have several strengths and limitations. To the best of knowledge, it is the first to provide a thorough analysis of the effects of *Moringa* on anthropometric measures and blood pressure. However, some limitations should be noted. The findings should be interpreted cautiously due to limitations in causal inference. Furthermore, the heterogeneity in participants' health conditions and the small sample sizes of the included studies limit the interpretation of the findings. The variability in outcome measurement protocols (e.g., blood pressure and BMI) will also contribute to heterogeneity. Additionally, it should be highlighted that meta‐analytic significance does not necessarily equate to clinical relevance, and observed subgroup effects (e.g., specific dose ranges or BMI categories) may reflect chance findings or residual confounding, particularly when derived from small trials or multiple arms of the same study. In addition, due to insufficient data in some studies, a dose–response analysis could not be conducted. Publication bias assessments were underpowered due to the limited number of studies; therefore, null findings and bias estimates should be interpreted with caution and supported by visual inspection of funnel plots. Given these limitations, future research should prioritize well‐powered, multicenter, preregistered RCTs using standardized *Moringa oleifera* formulations, clearly defined dosing regimens, longer and more uniform follow‐up periods, and consistent outcome measurement protocols, with stratification by baseline health status (e.g., hypertension, BMI) and medication use. Future reviews should apply more restrictive inclusion criteria or prespecified subgroup analyses and use meta‐regression and sensitivity analyses to better address heterogeneity, assess dose–response relationships, and improve the robustness and generalizability of conclusions.

## 5 Conclusion

Our systematic review and meta‐analysis of RCTs found that *Moringa oleifera* significantly improved blood pressure but not body weight or BMI. SBP decreased in adults consuming 5–10 g/day of *Moringa*, particularly in individuals with a BMI under 29.9 and those treated for more than 4 weeks. Additionally, *Moringa* intake was associated with a notable reduction in DBP among participants taking < 10 g/day, overweight individuals, and those who supplemented for over 4 weeks. Further studies are needed to investigate the effects of *Moringa oleifera* intake on blood pressure.

## Author Contributions

**Mahnoush Mehrzad Samarin:** writing – original draft. **Fatemeh Sheikhhossein:** writing – original draft. **Amir Hossein Khalilkhaneh:** writing – original draft, methodology. **Shadi Ghaemi:** writing – original draft. **Amin Abbasi:** writing – original draft. **Hadi Bazyar:** writing – original draft. **Farhang Djafari:** writing – original draft. **Zahra Dianati:** writing – review and editing, writing – original draft. **Seyed Mojtaba Khodashenas:** writing – original draft. **Gholamreza Askari:** writing – original draft. **Mohammad Reza Amini:** writing – original draft, conceptualization, data curation, supervision.

## Funding

Financial support for conception, design, data analysis, and manuscript drafting was provided by the Nutrition and Food Security Research Center, Isfahan University of Medical Sciences, Isfahan, Iran (no. 2404291).

## Ethics Statement

The authors have nothing to report.

## Consent

The authors have nothing to report.

## Conflicts of Interest

The authors declare no conflicts of interest.

## Supporting information


**Table S1:** PRISMA checklist.

## Data Availability

The data that support the findings of this study are available from the corresponding author upon reasonable request.
